# Supplementary material for: Predictors of Quality of Life in HIV-Infected Persons from Mozambique: The Dual Role of Schooling
Source: Infect Dis Rep. 2023 Jul 10;15(4):392–402. doi: 10.3390/idr15040040 (PMC10366898; doi:10.3390/idr15040040)
Supplement: Supplementary file 1 [file idr-15-00040-s001.zip › idr-2435139-supplementary.pdf]

Table S1

Clinical characteristics of participants

Clinical variables		N	%
**Clinical profile**			
	HIV1	352	100
	HIV2	-	-
**Transmission**			
	Sexual	23	6.6
	Blood	18	5.1
	Unknown	310	88.3
**Viral load**			
	< 50	304	86.4
	51-500	28	8.0
	501-3000	8	2.3
	3001-10000	4	1.1
	10001-30000	5	1.4
	>30000	2	0.6
**Secondary effects from treatment**			
	Fatigue	15	4.3
	Nausea and vomiting	3	0.9
	Pain	1	0.3
	Physical transformations	1	0.3
	Multiple effects	6	1.7
	Others	27	15.1
	No secondary effects	299	84.9
**Interruption of treatment**			
	yes	13	3.7
	no	338	96.3
**Reason to interrupt**			
	Secondary effects	2	0.6
	Lack of information	4	1.1
	Loss of patient's card	3	0.9
	Other reasons	5	1.4
**Change in treatment**			
	yes	41	11.6
	no	311	88.4
**Reasons to change**			
	Secondary effects	3	0.9
	High viral load	11	3.1
	Other reasons	13	3.7
	Unknown	14	4.0
**Other treatments**			
	yes	2	0.6
	no	348	99.4
